# Report of the Diseases of Edinburgh, for June 1808

**Published:** 1808-08

**Authors:** John Roberton

**Affiliations:** Surgeon


### *Report of the Diseases of Edinburgh, for June* 1808; *by* Mr. John Roberton, *Surgeon.*

This month commenced with heavy falls of rain, which, however, only continued at irregular periods for two or three days. These were followed by fogs, probably in consequence of the prevailing easterly winds; but by the end of the first fourth of the month, the weather, particularly during the day, became very warm and pleasant. Immediately after sun-set, heavy dews uniformly prevailed, which, till sun-rise the following morning, contributed very much to refresh the verdure that had, from the great heat of the day, become parched, and but for these dews would have been much retarded in its growth. Toward the end of the month these dews abated, and the weather was delightful.

During the whole month the thermometer was variable, and the barometer, for the most part, stood high.

The diseases of this month have neither been very numerous nor very severe. No epidemic has existed that seems to have proved destructive of human life in any remarkable degree.

Ophthalmia, which had in a great measure subsided, became much worse very early in the month, and for the most part of it continued excessively troublesome to many. I have used various external applications for the alleviation of the uncommon pain accompanying it; and these applications, though seemingly different in their nature, and applied to patients apparently of a very different habit of body, were all, for the first few hours, productive of relief. Indeed, the present form of this disease can scarcely be treated by any general rule. In the course of a very short period of time, I have found it necessary, in the same patient, to apply for a few hours solutions of the vitriolic preparations, or of the preparations of lead; and in the same day, perhaps, or the following, for the same patient, I have found it requisite to order the eye to be bathed with laudanum or with ardent spirits, or anointed with a strong ointment of the red oxyd of mercury; and all of these, in their turn, were productive of the most perfect relief; but a continuance of them always did harm. The rapid changes therefore from one to another of these substances, which I was obliged to make, was in consequence of their producing very different effect to what they did when first applied.

A sort

A sort of febrile complaint has prevailed during the month, particularly toward the end of it, of a teizing nature. The patients, for the most part, have not found it necessary to be confined to bed; but the gradual though slow exhaustion which their strength suffered, rendered them very unhappy. Most of the patients whom I have seen in this state, have recovered more quickly and more completely, by leaving the city and taking up their residence a mile or two in the country, than by almost any medicines which I could administer for their relief.

The susceptibility to inflammatory complaints seems very great in this part of the world at every period of the year. During the wet, cold months of winter, they are certainly more frequent in their occurrence, as well as more fatal in their consequences, than at any other period; and perhaps few winters have more clearly proved the truth of this observation, at least for many years past, than our last winter in this place. (See Reports for that time.) Since then, inflammatory diseases have occasionally made their appearance; and although, in general, they have never assumed such virulence as we observed among them during the winter, yet they have been sufficiently severe and numerous to constitute a very great proportion of those complaints which have made a very formidable appearance in the medical practitioner's list.

Peumonia and inflammation of the bowels still continue to prevail in many instances with considerable severity. Among those whose constitutions are not otherwise broken down by disease, bleeding or blistering, with saline purgatives, almost invariably effect a cure. With those, however, who are debilitated by age, enfeebled by vicious habits, or broken down by disease, the first of these applications must almost always be avoided, as it is seldom if ever serviceable, and often highly injurious. Dropsy, in one or other, or almost in every form, is the consequence of such treatment in the above habits; and when once induced in them, can, I may venture to say, never be removed. Alleviation may sometimes be obtained, but this is always very deceitful, and of short duration. The medical attendant, therefore, cannot be too careful in discriminating, with great caution, the habit of body, previous manner of living, &c. of patients affected with the above complaint, before employing venesection, even although several symptoms indicate the propriety of such practice. If the habit of body have been previously much injured, injured, greater benefit, for the most part, will be obtained by blistering than by bleeding, or any similar method which may greatly reduce the general system.

No. 4, *St. James's Street.*

